# Mapping and Predicting Patterns of Chinese Adolescents’ Food Preferences

**DOI:** 10.3390/nu11092124

**Published:** 2019-09-06

**Authors:** Shaojing Sun, Jinbo He, Xitao Fan

**Affiliations:** 1 School of Journalism, Fudan University, Shanghai 200433, China; 2 School of Humanities and Social Science, the Chinese University of Hong Kong (Shenzhen), Shenzhen 518172, China

**Keywords:** latent class analysis, food preference, Chinese, adolescents

## Abstract

This study aimed to examine the patterns of, as well as the predictors for, Chinese adolescents’ food preferences. Using the national data of the China Health and Nutrition Survey (CHNS), we analyzed the data of 697 adolescents in the age range of 12 to 17 years. Latent class analysis revealed four types of food preferences: *varied diet* (37.09%, *n* = 254), *avoiding vegetables* (19.69%, *n* = 131), *low appetite* (7.56%, *n* = 50), and *healthy diet* (35.66%, *n* = 222). Major predictors for food preferences included demographic variables (e.g., gender, urban versus rural residence), nutrition knowledge, preference for activities, and social attitudes. Results did not show any significant differences in BMI *z*-scores among the four latent classes. However, there were significant differences in the number of sleeping hours among the classes.

## 1. Introduction

Food preference refers to the degree of liking or disliking food [1]. Ample evidence has indicated that food preference is closely related to a variety of physical health outcomes, such as micronutrient inadequacy [2], obesity [3], cardiovascular disease [4], and cancer [5]. Given the rising rates of obesity and vascular disease [6] and the poor dietary choices in China [7], promoting healthy diet (e.g., fruit and vegetable intake) has become a vital issue for public health [8].

It has been suggested that one important way to improve dietary quality at the population level is to identify behaviors and related characteristics affecting one’s adherence to dietary recommendations and guidelines [9]. As such, it would be meaningful to explore potential predictors or risk factors (e.g., food preference) associated with unhealthy dietary patterns. Despite being related to each other, past research has distinguished between dietary patterns and stated preferences for food [10]. The former speaks to one’s actual food consumption and dietary history, whereas the latter reflects the underlying attitude and motivation. Our study focused on food preferences, and it differed fundamentally from recent studies on dietary patterns (e.g., Zhen et al. [11]). Specifically, the food preferences were measured with attitude-related questions (e.g., how much do you like fast food?) in our study, while the dietary patterns were measured with questions tapping into children’s actual behavior or dietary history (e.g., “whether you had rice, noodles, candy, milk in the past three days”) in Zhen et al. [11]. Differentiation of dietary patterns and preferences is particularly important for children and adolescents, as their actual food consumption is often contingent on their parents’ decision [12].

Many studies have indicated that food preference is a complex phenomenon, as it is premised on a range of psychological, social, and cultural factors [13,14]. Pearson et al. [13] found that individual habits (e.g., eating while watching TV), social environment (e.g., parental pressure to eat), and physical environment (e.g., availability of fruits and vegetables at home) together influenced young adolescents’ preference for consumption of fruits and vegetables. Verstraeten et al. [15], in a sample of 784 school-age Ecuadorian adolescents, found that both individual factors (e.g., perceived benefits of food) and environmental factors (e.g., school support and parental permissiveness) significantly affected one’s eating behaviors (e.g., vegetable intake, unhealthy snacking). Examining the relationship between food consumption and physical activity, Choi and Ainsworth [16] found that active men consumed more grain products, fruits, and vegetables than did the sedentary people. On the other hand, active women tended to consume more legumes and vegetables than did the sedentary ones. Fussner, Luebbe, and Smith [17] showed that disordered eating symptoms were significantly associated with one’s sensitivity to social reward and social punishment. More succinctly, de Ridder et al. [18] concluded that individual factors (e.g., intentions, self-regulatory skills) and social/environmental factors (e.g., social norms, availability) are the most important determinants of a healthy diet. 

Adolescence, an important transition stage from childhood to adulthood, entails dramatic biological, emotional, and cognitive changes [19]. With these changes, adolescents are particularly susceptible to inadequacies of nutrients [20]. The effects and consequences of dietary patterns during this important transition period have received considerable attention in the research literature. Movassagh et al. [21] identified five types of dietary patterns (i.e., “Vegetarian-style”, “Western-like”, “High-fat, high-protein”, “Mixed” and “Snack”) among adolescents. Furthermore, the vegetarian-style dietary pattern during adolescence had a positive long-term impact on one’s bone health. 

In addition to the dietary issues associated with adolescents, researchers have been paying more attention to the relationships between dietary factors and other related health issues (e.g., body weight, sleep). For the relationship between adolescents’ dietary factors and weight change, Laska et al. [22], in a sample of adolescents in Minnesota, found that their diet soda intake was positively related to BMI among females, but not among males. Also, past research supported the linkage between diet and sleep quality, although the mechanism underlying the linkage is not always clear. Peuhkuri, Sihvola, and Korpela [23] summarized past studies and contended that food intake could affect sleep. They pointed out that a balanced and varied diet (e.g., rich in vegetables, fruits, whole grains, low-fat protein) could improve sleep, because such diets might stimulate the synthesis of serotonin and melatonin that were conducive to better sleep. Similarly, St-Onge, Mikic, and Pietrolungo [24] noted that past studies, though mixed and focusing on short-term effects, tended to support the conclusion that some foods (e.g., fish, fruits, vegetables) were sleep-promoting. St-Onge et al. [24], however, called for clinical studies as to exploring the long-term effects of dietary patterns on sleep duration and quality. Notably, recent research has begun to examine the dynamic relationship between diet and sleep duration, as well as the implications for weight-related outcomes, obesity, and other chronic diseases [25]. 

Given that nutrition intake is a cultural and biological process, rather than a mere physiological and biochemical process [26], prior findings on food consumption in western cultural context might not be readily generalizable to Chinese adolescents. Moreover, to date, little research has been conducted to explore Chinese adolescents’ food preferences and the related predictors and health risks thereof [27,28]. For instance, Shi et al. [28] found that more than half of Chinese students reported liking for Western-style fast foods (hamburgers, soft drinks and chocolate). Nonetheless, the studies of Shi et al. [28] and Deng [27] mainly focused on the influences of SES (socioeconomic status) on adolescents’ food preferences. As a matter of fact, despite being potentially predictive of Chinese adolescents’ food preferences, a broad range of psychological, social, and cultural factors remain understudied. 

Overall, past research on adolescents’ food preferences mainly focused on the western cultural context. In contrast, food consumption of youngsters is particularly understudied in Asian cultures such as China. In the present study, using a national dataset and the analytic approach of Latent class analysis (LCA) model, we aimed to explore the typology and potential predictors/correlates (e.g., demographics, nutrition knowledge, preference for physical activities, social attitudes, sleep hours) of food preferences.

## 2. Methods

### 2.1. Data Description

We used the publicly available data of the “China Health and Nutrition Survey” (CHNS), which was sponsored and designed by the Carolina Population Center at the University of North Carolina at Chapel Hill and the National Institute of Nutrition and Food Safety at the Chinese Center for Disease Control and Prevention. The survey focused on health and nutritional issues of Chinese population. An important goal of the project was to investigate the impact of changes, occurring at community, household, and individual levels, on one’s health/nutrition behavior and outcomes. 

The first wave of the survey was in 1989, and since then, there have been ten waves of data collection. In this study, we used the most recently released wave of data collected in 2011, covering 289 communities and 5923 families, with 15,725 participants in total. Data for major variables in the present study are available only for those above 12 years old. With this restraint, we identified a total of 697 adolescents between 12 and 17 years old as the sample of this study. 

Of the 697 participants, 51.5% were male and 48.5% were female. The average age was 14.25 years old with a standard deviation of 1.65. As for education, 35.6% of the adolescents were elementary school students and 48.8% were middle/high school students. Also, 48.5% of the participants were living in urban regions, whereas 51.5% were living in rural regions at the time of survey. Table 1 presents the descriptive statistics for the total sample. 

### 2.2. Instruments

**Food preferences**. Respondents were asked to describe how much they like (“dislike very much,” “dislike,” “neutral,” “like,” “like very much,” or “does not eat this food”) five kinds of food: (1) fast food (KFC, pizza, hamburgers, etc.); (2) salty snack foods (potato chips, pretzels, French, fries, etc.); (3) fruits and vegetables, and (4) soft drinks and sugared fruit drinks. According to Collins and Lanza (2010) [29], Likert-scale responses are often categorized into binary responses in latent class analyses for ease of interpretation. Thus, the responses to each question for food preferences were collapsed into two categories (‘like’ and ‘dislike’). Specifically, responses of both “like very much” and “like” were grouped into one category of “like,” whereas responses of “dislike very much,” “dislike,” “neutral,” or “does not eat this food” were grouped into the other category of “dislike.” This approach is consistent with the research practice in previous research (e.g., Hardigan & Sangasubana [30]).

**Background variables**. Data on social economic status (SES) and other demographic variables were collected from the participants. For the present study, we focused on the following variables: gender, age, education, residence (urban rural rural), BMI, and hours of sleep per day. For gender, male was coded as 1 and female was coded as 2. Age was calculated by subtracting the year of birth from the time of interviewing. Education was classified into five categories ranging from primary school to college. Hours of sleeping was measured by a single item asking for the total hours spent on sleeping in daytime and at night. The BMI was derived from self-reported weight and height [31]. As suggested by Cole, et al. [32], BMI *z*-score is the optimal measure of adiposity on a single occasion (i.e., not longitudinal change), and so we used the BMI *z*-score calculated via the R package childsds [33].

**Nutrition knowledge**. Respondents’ knowledge of nutrition was measured by nine items on 5-point Likert scale. The instruction asked the respondents about the degree to which they would agree with each of the nine statements. Some example items were: “choosing a diet with a lot of fresh fruits and vegetables is good for one’s health,” “choosing a diet with a lot of staple foods ‘rice and rice products and wheat and wheat products’ is not good for one’s health,”, and “consuming a lot of animal products daily (fish, poultry, eggs and lean meat) is good for one’s health.”

**Preference for activities**. Six items were used to measure participants’ preference for activities. Response options ranged from 1 (dislike very much) to 5 (like very much). Listed activities include walking/Tai Chi, sports (ping pong, badminton, tennis, soccer, basketball, volleyball), body building, watching TV, playing computer/video games/surfing the internet, and reading.

**Social attitudes**. Four items were used to assess the respondents’ social attitudes on a 1–4 Likert scale. Participants were asked of the degree to which they care about the following: (a) being praised by their parents, (b) being liked by friends, (c) looking fashionable, and d) achieving high scores in school. 

**Sleeping**. One item was used to measure an adolescent’s sleeping duration. The item reads as “including daytime and nighttime, how many hours do you typically spend on sleeping each day?” 

### 2.3. Data Analysis

Latent class analysis (LCA) is a technique often used for identifying “latent” (i.e., unobserved) subgroups of individuals with distinct patterns of responses [34]. Previous studies have shown the advantages of using LCA to identify distinct patterns of food preferences. Specifically, the technique allows researchers to identify different “classes” (i.e., groups) of individuals, with members within the same group being relatively similar and those across groups being relatively dissimilar in food preferences. Once these “latent classes” are statistically identified, it is possible to examine the unique characteristics of each class [35]. Furthermore, identifying subgroups via LCA is especially useful for designing prevention/treatment programs targeting specific groups with higher level of health risk [36]. 

Latent class analysis was conducted using Mplus version 8.3 [37] with the robust maximum likelihood estimator (MLR). A large number of starting values (500 random sets of start values with 100 best solutions retained) were used to explore the true highest log likelihood value [38]. Comparing models with 1 to 5 profiles, we searched for the optimal number of latent profiles through the following fit indicators: Akaike Information Criterion (AIC), Bayesian Information Criterion (BIC), Sample Size Adjusted BIC (SABIC), Bootstrapped Likelihood Ratio Test (BLRT), Lo-Mendell-Rubin Adjusted Likelihood Ratio Test (LMRT), and Entropy. Generally speaking, lower values of the AIC, BIC, and SABIC suggest a better model fit, whereas higher values of entropy suggest better quality of classification. The two indices of LMRT and BLRT w employed to compare the discrepancy between two models (*k* classes versus *k*-1 classes), with statistical significance suggesting that the model with *k*-1 classes is preferable. However, these fit indices should not be treated as iron rules for comparing models; rather, researchers should take into consideration practical interpretability and theoretical implications in the model-selection process [39,40]. 

After identifying the optimal number of classes in the sample, we explored the characteristics of the profiles by adding covariates into the LCA model, in light of the recommended three-step approach [41]. The number of predictors was also controlled for all the steps of the statistical procedure. Specifically, conducting multinomial logistic regressions, we investigated the predictive power of multiple variables (demographic variables, personal knowledge of nutrition, preference for activities, and one’s social attitudes) for the class memberships. Furthermore, we examined whether adolescents in different classes would differ in BMI and in hours of sleeping. For the very small amount of missing values, we used the list-wise deletion method to handle the missing data, as the percentage of missing values on major variables was generally less than 2%, which could be considered inconsequential in reference to the general standard of 5% [42].

## 3. Results

### 3.1. Latent Class Analysis

The values of the fit indicators for model comparison in LCA are reported in Table 2. Specifically, we listed the model fit information for five different models, ranging from 1 class to 5 class. The LL denotes the likelihood ratio of each model, whereas AIC, BIC, and SABIC serve as fit indices of the models. Typically, the smaller the three aforementioned fit indices, the better model fit. The LMRT and BLMRT were conducted to compare two nested models, with statistical significance suggesting that the compared two models are significantly different from each other. Entropy indicates the accuracy of classification, with a larger value indicating higher classification accuracy. The mixing ratio represents the proportion of each latent class in the population. The results showed that the values of the AIC, BIC, and SABIC deceased with the number of latent classes increasing from 1 to 4 incrementally. However, with the number of classes increasing from 4 to 5, values of these indices did not decrease anymore. Furthermore, in terms of BLRT, the *p* value for the 5-class model was greater than 0.05 (i.e., non-significant), indicating that the 5 class model was not better than the 4 class model. The *p* values of the LRT showed the 4-class model was better than the models with a smaller number of classes. The values of Entropy, ranging from 0.67 to 0.86, also supported the superiority of the 4-class model over the alternative ones.

### 3.2. Characteristics of Latent Classes

**Figure 1:**
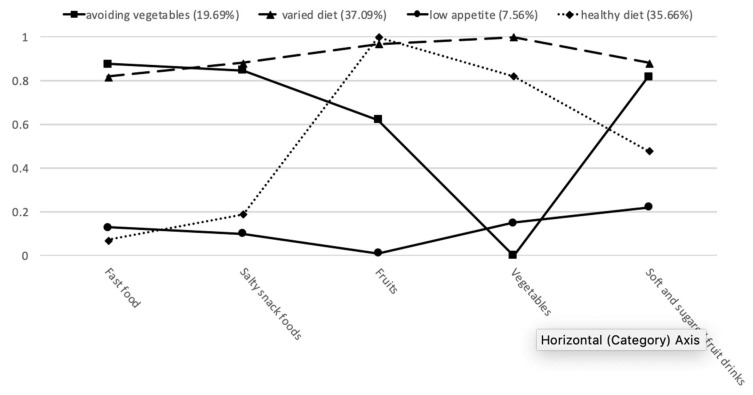
Description of the patterns of food preferences. *x*-axis = indicators of food preferences (i.e., fast food, salty snack, fruits, vegetables, soft drinks/sugared fruit drinks); *y*-axis = probability of a “Like” response to each food preference item conditional on latent status membership.

Figure 1 and Table 3 show the patterns of scores on food preference items for each identified class. Specifically, presented in Table 3 are the probabilities of endorsing each item by the respondents classified into a particular class. Figure 1 presents the profiles of each class for the 4 class solution. Class 1, accounting for 19.69% of the sample, was labeled *avoiding vegetables*, because participants in this class showed strong preferences for all types of food except vegetables. Class 2, accounting for 37.09% of the sample, was labeled *varied diet*, as participants in this class were characterized by strong preferences for all five types of food. Class 3, accounting for 7.56% of the sample, was labeled *low appetite*, as participants in this class showed weak preferences for all five types of foods or drinks. Class 4, accounting for 35.66% of the sample, was labeled *healthy diet*, because participants in this class had strong preferences for healthy food types (i.e., fruits and vegetables) but weak preferences for unhealthy food types (i.e., fast food, salty snack food, and soft drinks).

### 3.3. Predictors of Latent Class Membership

To explore how different variables were related to class membership, we used multinomial logistic regression as follow-up analysis to LCA, and examined how different sets of variables could be predictive of the membership in one of the four latent classes. Because multiple post-hoc group comparisons of LCA could lead to inflated overall Type I error rate, we used Bonferroni method for better control of Type I error. To balance statistical power and the Type I error control, we set the overall significance level at 0.10. Thus, the Bonferroni-corrected significance level under each of six comparisons, as shown in Table 4, was set at α = 0.10/6 ≈ 0.02.

Food preference and demographic variables. Among the background variables, compared to boys, girls were less likely to be in the class of *low appetite* (*OR* = 0.52, *p* < 0.02). Rural participants were more likely to be in the class of *healthy diet* than urban participants (*OR* = 1.62, *p* < 0.02). The higher the education, the less likely one would be in the class of *avoiding vegetables*. Compared to younger ones, older adolescents showed a higher likelihood of being in the class of *low appetite* (*OR* = 1.24, *p* < 0.02) and lower likelihood of being in the class of *avoiding vegetables* (*OR* = 0.77, *p* < 0.02). 

Food preference and nutrition knowledge. Out of the 12 variables for nutrition knowledge, nine factors showed statistically significant predictive effects on adolescents’ food preferences. Those believing that eating fruits and vegetables are good for health were less likely to be in the class of *low appetite* than others. Those favoring eating sugar had a high likelihood to be in the classes of *low appetite* or *avoiding vegetables* (*OR* = 1.47~1.69, *p* < 0.02), but low likelihood to be in the class of *healthy diet* (*OR* = 0.48~0.56, *p* < 0.01). Those who favor dairy food or milk were less likely to be in the class of *low appetite* (*OR* = 0.49, *p* < 0.02), but were more likely to be in the class of *avoiding vegetables* (*OR* = 1.74, *p* < 0.02) or *healthy diet* (*OR* = 1.67, *p* < 0.02). Those who favor animal products were less likely to be in the class of *healthy diet* (*OR* = 0.61~0.75, *p* < 0.02). Results also showed that those favoring beans were less likely to be in the classes of *low appetite* (*OR* = 0.51, *p* < 0.02) or *avoiding vegetables* (*OR* = 0.67, *p* < 0.02) than in the class of *varied diet*. 

Food preference and activity preference. Walking was associated with lower likelihood to be in the class of *avoiding vegetables* (*OR* = 0.71, *p* < 0.02), whereas sports was associated with lower likelihood of being in the class of *low appetite* (*OR* = 0.71, *p* < 0.02) but higher likelihood of being in the class of *healthy diet* (*OR* = 1.41, *p* < 0.02). Body building did not emerge as a significant predictor of any latent class. Watching TV was predictive of lower likelihood of being in the classes of *low appetite* (*OR* = 0.41, *p* < 0.001) and *healthy diet* (*OR* = 0.51, *p* < 0.001), but predictive of higher likelihood of being in the class of *avoiding vegetables* (*OR* = 2.24, *p* < 0.001). The effect of playing games showed a pattern similar to that of watching TV. Interestingly, preference for reading predicted lower likelihood of being in the class of *low appetite* (*OR* = 0.57, *p* < 0.01) but higher likelihood of being in the classes of *heathy diet* (*OR* = 2.09, *p* < 0.001) and *avoiding vegetables* (*OR* = 1.51, *p* < 0.02). 

Food preference and social attitudes. High social attitudes (i.e., caring about parents’ compliments, friends’ likes, being fashionable, and school performance), in general, predicted higher likelihood of being in the class of *varied diet* than in the classes of *low appetite* (*OR* = 0.38~0.41, *p* < 0.01), *avoiding vegetables* (*OR* = 0.72~0.74, *p* < 0.02), and *healthy diet* (*OR* = 0.58~0.66, *p* < 0.02). But, between classes of *avoiding vegetables* and *low appetite*, higher social attitudes signified higher probability of belonging to the former group (*OR* = 0.93~0.96, *p* < 0.02). 

### 3.4. Latent Class Membership, BMI and Sleeping Hours

The results showed no statistically significant differences of BMI z-scores among the four latent classes. Interestingly, there was significant difference of sleeping hours among the classes ($\chi^{2}$(3) = 8.35, *p* = 0.039). Specifically, adolescents in *healthy diet* group (*M* = 8.47, *SE* =0.07) reported longer hours of sleeping than those in *low appetite* group (*M* = 8.10, *SE* = 0.14, $\chi^{2}$(1) = 6.12, *p* < 0.01) and those in *avoiding vegetables* group (*M* = 8.24, *SE* = 0.09, $\chi^{2}$(1) = 4.34, *p* = 0.037). Although adolescents in *healthy diet* group (*M* = 8.33, *SE* = 0.07) reported longer sleeping hours than those in *low appetite* and *avoiding vegetables* groups, the differences were not statistically significant. 

## 4. Discussion

The present study used a national dataset from China and explored adolescents’ food preferences and factors related to such preferences. To our knowledge, our study is the first one using the approach of latent class analysis (LCA) to study Chinese adolescents’ food preferences. This method has proven to be very useful to identify latent groups, and it provides a novel perspective on prevention and treatment. At the theoretical level, our study contributes to the differentiation between food preference and actual food consumption. Food preferences should be conceptualized as an attitude-related construct instead of a behavior such as actual food consumptions. In this regard, in spite of the studies focusing on the patterns of food consumptions among Chinese adolescents (e.g., [11]), our study is significantly different due to the fact of its focus on food preferences, which have been shown to be related to eating behaviors and psychological well-being We identified four types of food preferences: *avoiding vegetables*, *varied diet*, *low appetite*, and *healthy diet*. Adolescents in the classes of *varied diet* and *healthy diet* accounted for more than 70% of the total respondents. It seems that vegetables and fruits were the major types of food that distinguished respondents’ food preferences. Soft drink and fast food, however, were favored options by a large proportion of adolescents. 

## 5. Significant Findings and Implications

Our study found that demographic variables played a significant role in predicting adolescents’ food preferences. Girls were more likely to be in the class of *healthy diet* than boys. Although the present study was conducted in the Chinese cultural/social context, the findings echoed those of prior studies conducted in western cultural/social contexts. For example, Caine-Bish and Scheule [43] surveyed American children and reported that food preferences differed across gender. Furthermore, the gender difference varied among elementary, middle, and high school students. Their findings revealed that boys preferred meat, fish, and poultry food, whereas girls preferred fruits and vegetables. Considering the consistency of gender difference in food preference across cultures, practitioners may take into account the role of gender while designing dietary interventions for Chinese adolescents.

One interesting finding speaks to the impact of residence status. In China, people’s residence is roughly classified into two types: urban residence vs. rural residence. Urban residents rarely have resources for farming or growing crops or vegetables. In contrast, rural residents are more likely to have access to rich supplies of vegetables. Such a difference may explain why adolescents of rural residence were more likely to be in the class of *healthy diet*, but less likely to be in the class of *avoiding vegetables* than those of urban residence. It is plausible that those growing up in rural regions had easy access to vegetables and formed the habit of eating vegetables over time. As prior studies have shown [44], accessibility of food is a significant predictor of food preference. Thus, health education and promotion programs in China should take into consideration the differences between urban and rural residence.

Regarding the impact of nutrition knowledge, an interesting finding is that nutrition knowledge appears to influence one’s food preferences. More specifically, our results showed that one’s nutrition knowledge about sugar, animal products, and dairy food was predictive of his/her food preferences. Research has shown that nutrition knowledge is closely related to food intake, especially for healthy eating. Therefore, improving nutrition knowledge could be a target for health education campaigns for promoting healthy eating [45]. On this note, school-based nutrition education for Chinese adolescents could play an important role in promoting healthy eating. 

As for the relationship between activity preference and food preferences, the finding about watching TV was in line with prior findings that heavy TV watching may increase children’s preferences for unhealthy foods (e.g., high carbohydrate and high fat foods) [46,47]. Not surprisingly, the amount of computer-game playing also emerged to be a significant predictor of food preferences. Past research has shown that both watching TV and playing computer games increased adolescents’ cravings for unhealthy snacks and drinks [48]. Our finding speaks to the common nature of the two media activities, which are sedentary and addictive [49,50]. A bit puzzling is the relationship between reading and food preferences, with reading being associated with higher likelihood of being in the class of *healthy diet* as opposed to the classes of *low appetite* or *avoiding vegetables*. One plausible explanation is that those enjoying reading might have learned more about nutrition and healthy dietary practice. 

The linkage between social attitudes and food preferences may attest to cultural/social influences. Interestingly, we found that those who cared about parents’/friends’ views and one’s own social images were more likely to be in the class of *varied diet*, as opposed to be in the other three classes. It is likely that those with higher scores of social attitudes are adaptable to different life contexts, and hence more readily acceptable to varying food choices. 

Our study did not reveal any significant differences in BMI z-scores among the four classes of individuals. This finding, however, is in line with some previous studies showing that food preferences were not related, or only marginally related, to BMI [3,51]. Food preferences could be related to nutrition status, but not necessarily to BMI [52]. 

Finally, the study revealed that adolescents with a high variety of food consumption (i.e., *varied diet*) or healthy food consumption (i.e., *healthy diet*) had longer sleeping hours than those with a low variety of food consumption (i.e., *low appetite*) or unhealthy food consumption (i.e., *avoiding vegetables*). Past research has provided empirical evidence on the linkage between sleep duration and dietary behaviors [53]. For example, Franckle et al. [54] reported that insufficient sleep among children and adolescents was linked to unhealthy dietary behaviors (e.g., decreased vegetables consumption). Future dietary interventions among Chinese adolescents may consider incorporating strategies for improving adolescents’ sleep duration. 

## 6. Limitations and Future Directions

The present study has several limitations. First, although we used a national sample of adolescents, the sample size may not be large enough to generalize the findings to the general adolescent population. Second, the measurement of food preferences and nutrition knowledge may not be satisfying due to the small number of items; thus, we highly encourage future researchers to conduct similar studies by using more standardized and well-established instruments (e.g., the General Nutrition Knowledge Questionnaire [55], the Adult Eating Behaviour Questionnaire [56,57]). In reality, there is a much larger variety of food that adolescents consume in their daily lives. Future research should consider more comprehensive instrument(s) to assess food preference. Third, social factors could be much broader, relating to family, community, and cultural contexts. The current study only assessed participants’ general attitudes toward parents, friends, and school. Future studies should consider the multidimensional nature of social environment and explore more potential factors in this regard. Adolescence is a vital stage in one’s formation of outlooks about life and the world. It would be important to understand what habits and preferences for food will carry over into adulthood and have a lasting impact. Research has shown that adolescents’ food preference is related to their general health and well-being. Researchers, if possible, should look at the potential consequences, both proximal and distal, of different food preferences. 

## 7. Conclusions

Our study identified four different types of food preferences among Chinese adolescents, with each presenting its own unique characteristics. We further revealed that various factors (e.g., gender, residence, and nutrition knowledge) were closely related to the types of food preferences. Future dietary interventions (e.g., school-based nutrition education) should target specific groups of Chinese adolescents with attributes known to be associated with low vegetable or high fast food consumption. 

## Figures and Tables

**Table 1:** Descriptive statistics for the total sample (*n* = 697).

	Mean *±* SD/% (*n*)	Min-Max	Skewness	Kurtosis
Gender (male = 1)	51.5% (359)	1–2	0.06	−2.01
Education (primary school = 1)	35.6% (248)	1–5	0.64	0.73
Residence (urban = 1)	48.5% (338)	1–2	−0.06	−2.00
Age	14.25 ± 1.65	12–17	0.18	−1.12
BMI	19.61 ± 3.44	13.24–37.99	1.05	1.79
Sleeping hours	8.37 ± 1.04	5–12	0.23	0.59
Dietary knowledge				
Choose fruits/vegetables	3.72 ± 0.83	1–5	−1.14	1.21
Eating sugar	2.15 ± 0.62	1–5	1.23	2.81
Eating a variety of food	3.73 ± 0.74	1–5	−1.38	2.15
Diet high in fat	2.11 ± 0.69	1–5	1.18	2.39
Diet of staple food	3.15 ± 0.96	1–5	−0.29	−0.95
Diet of animal products	2.64 ± 0.93	1–5	0.51	−0.82
Reducing fatty meat	3.69 ± 0.82	1–5	−1.26	1.33
Milk and dairy products	4.00 ± 0.54	1–5	−1.87	9.95
Beans and bean products	3.99 ± 0.53	1–5	−1.77	9.92
Preference for activities				
Walking	2.54 ± 0.89	1–5	0.61	0.05
Sports	3.69 ± 1.07	1–5	−0.44	−0.78
Body building	2.86 ± 0.98	1–5	0.49	−0.40
Watching TV	4.05 ± 0.86	1–5	−0.87	0.82
Playing games	3.78 ± 1.09	1–5	−0.52	−0.79
Reading	3.52 ± 0.94	1–5	−0.27	−0.61
Life attitudes				
Praise from parents	2.32 ± 0.77	1–4	0.35	−0.14
Being liked by friends	2.34 ± 0.85	1–4	1.68	10.47
Look fashionable	2.35 ± 0.82	1–4	1.13	6.47
Achieve high scores in school	2.34 ± 0.86	1–4	1.67	10.38

*Notes*: The amount of missing data varies across variables.

**Table 2:** Fit indices and class proportions for the 1 to 5 class models.

Classes	LL	AIC	BIC	SABIC	LMRT *p*-Value	BLRT *p*-Value	Entropy	Mixing Ratio
1	−1987.80	3985.60	4008.04	3992.17	-	-	-	-
2	−1853.49	3728.98	3778.35	3743.42	<0.001	<0.001	0.67	0.45/0.55
3	−1793.25	3620.50	3696.79	3642.82	<0.001	<0.001	0.74	0.46/0.16/0.38
4	−1765.63	3577.25	3680.47	3607.44	<0.001	<0.001	0.84	0.37/0.08/0.20/0.35
5	−1761.94	3581.89	3712.03	3619.96	<0.05	>0.05	0.86	0.34/0.38/0.03/0.07/0.18

Notes: LL = the Log Likelihood; AIC = the Akaike Information Criterion; BIC = the Bayesian Information Criterion; SABIC = the Sample-Size Adjusted BIC; LMRT = the Lo-Mendell-Rubin Adjusted Likelihood Ratio Test.

**Table 3:** Item response probabilities for the four latent classes (avoiding vegetables, varied diet, low appetite, and healthy diet).

	Four Latent Classes of Food Preferences			
Types of Food	Avoiding Vegetables	Varied Diet	Low Appetite	Healthy Diet
	19.69% (*n* = 131)	37.09% (*n* = 254)	7.56% (*n* = 50)	35.66% (*n* = 222)
	Mean ± S.E.	Mean ± S.E.	Mean ± S.E.	Mean ± S.E.
Fast food	0.88 ± 0.05	0.82 ± 0.04	0.13 ± 0.07	0.07 ± 0.04
Salty snack food	0.85 ± 0.04	0.88 ± 0.04	0.10 ± 0.08	0.19 ± 0.05
Fruits	0.62 ± 0.05	0.97 ± 0.01	0.01 ± 0.39	1.00 ± 0.00
Vegetables	0.00 ± 0.00	1.00 ± 0.00	0.15 ± 0.06	0.82 ± 0.07
Soft/sugared fruit drinks	0.82 ± 0.04	0.88 ± 0.03	0.22 ± 0.08	0.48 ± 0.05

**Table 4:** Multinomial logistic regression predicting latent class membership as a function of predictors.

	Low Appetite versus Varied Diet			Avoiding Vegetables versus Varied Diet			Healthy Diet versus Varied Diet			Avoiding Vegetables versus Low Appetite			Healthy Diet versus Low Appetite			Healthy Diet versus Avoiding Vegetables		
	*B*	*SE*	*OR*	*B*	*SE*	*OR*	*B*	*SE*	*OR*	*B*	*SE*	*OR*	*B*	*SE*	*OR*	*B*	*SE*	*OR*
Gender	−0.64 *	0.35	0.52	−0.39	0.24	0.68	0.08	0.22	1.09	0.26	0.39	1.29	0.72	0.35	2.06	0.47	0.25	1.59
Residence	−0.01	0.34	0.99	−0.29	0.24	0.75	0.19	0.22	1.21	−0.28	0.37	0.76	0.20	0.34	1.22	0.48 *	0.25	1.62
Education	0.49	0.22	1.64	−0.02	0.16	0.98	0.28 *	0.14	1.32	−0.51 *	0.24	0.59	−0.22	0.22	0.80	0.29	0.16	1.34
Age	0.21 *	0.10	1.24	−0.05	0.08	0.95	0.09	0.07	1.09	−0.27 *	0.11	0.77	−0.13	0.10	0.88	0.14	0.08	1.15
Nutrition knowledge																		
Fruits/vegetables	−0.33 *	0.19	0.72	−0.08	0.14	0.92	0.01	0.14	1.00	0.24	0.21	1.28	0.33	0.20	1.39	0.08	0.15	1.09
Eating sugar	0.52 *	0.27	1.69	0.38 *	0.19	1.47	−0.21	0.19	0.82	−0.14	0.29	0.87	−0.73 **	0.27	0.48	−0.59 **	0.21	0.56
Eating variety	−0.47 *	0.22	0.63	−0.23	0.16	0.80	−0.06	0.16	0.94	0.24	0.23	1.27	0.41 *	0.21	1.51	0.17	0.16	1.18
Diet high in fat	0.30	0.24	1.36	−0.12	0.17	0.89	−0.28	0.16	0.76	−0.43	0.26	0.65	−0.58 *	0.24	0.56	−0.16	0.18	0.86
Staple food	−0.08	0.18	0.93	−0.04	0.13	0.96	0.04	0.12	1.04	0.03	0.20	1.03	0.19	0.18	1.13	0.09	0.14	1.09
Animal products	0.21	0.17	1.23	0.05	0.13	1.05	−0.28 *	0.13	0.75	−0.15	0.18	0.85	−0.49 **	0.17	0.61	−0.34 *	0.14	0.71
Reducing fatty meat	−0.35 *	0.19	0.70	−0.04	0.16	0.96	−0.06	0.14	0.94	0.32	0.22	1.37	0.29	0.18	1.34	−0.02	0.16	0.98
Milk and dairy products	−0.71 *	0.28	0.49	−0.15	0.22	0.86	−0.19	0.24	0.82	0.56 *	0.26	1.74	0.51 *	0.27	1.67	−0.04	0.22	0.96
Beans and bean products	−0.67 *	0.29	0.51	−0.40*	0.23	0.67	−0.18	0.24	0.84	0.27	0.28	1.31	0.49	0.30	1.64	0.22	0.23	1.25
Physical activities																		
Walking	−0.28	0.20	0.76	−0.34 *	0.14	0.71	−0.11	0.13	0.90	−0.06	0.22	0.94	0.17	0.20	1.19	0.24	0.15	1.27
Sports	−0.35 *	0.16	0.71	−0.05	0.11	0.95	−0.01	0.10	0.99	0.29	0.18	1.34	0.34 *	0.16	1.41	0.05	0.12	1.05
Body building	−0.39	0.21	0.68	−0.09	0.12	0.92	−0.10	0.12	0.90	0.30	0.23	1.35	0.28	0.21	1.33	−0.02	0.13	0.98
Watching TV	−0.90 ***	0.19	0.41	−0.09	0.16	0.91	−0.67 ***	0.16	0.51	0.80 ***	0.20	2.24	0.23	0.17	1.25	−0.56 ***	0.16	0.56
Playing games	−0.39 *	0.17	0.68	0.38 **	0.14	1.46	−0.26 *	0.10	0.77	0.77 ***	0.21	2.16	0.13	0.17	1.14	−0.64 ***	0.14	0.53
Reading	−0.56 **	0.18	0.57	−0.16	0.13	0.86	0.17	0.13	1.19	0.41 *	0.18	1.51	0.74 ***	0.17	2.09	0.33 *	0.13	1.39
Social attitudes																		
Praise from parents	−0.97 **	0.30	0.38	−0.30 *	0.15	0.74	−0.47 **	0.16	0.63	0.67 *	0.31	1.96	0.50	0.29	1.65	−0.17	0.16	0.84
Being liked by friends	−0.95 **	0.30	0.39	−0.29 *	0.14	0.74	−0.42 *	0.19	0.66	0.66 *	0.31	1.93	0.53	0.29	1.70	−0.13	0.17	0.88
Look fashionable	−0.89 **	0.29	0.41	−0.33 *	0.15	0.72	−0.55 **	0.16	0.58	0.56	0.30	1.74	0.34	0.29	1.40	−0.22	0.16	0.80
Achieve high scores in school	−0.96 **	0.32	0.38	−0.30 *	0.14	0.74	−0.42 *	0.19	0.66	0.66 *	0.33	1.93	0.54	0.31	1.72	−0.12	0.17	0.89

Notes: parents care—about parents’ praise; friends—care about being liked by friends; fashionable—like being fashionable; school—care about performance in school; SE: approximate standard error; OR = odds ratio; * *p* < 0.02, ** *p* < 0.01, *** *p* < 0.001.
